# Development of an interstitial cystitis risk score for bladder permeability

**DOI:** 10.1371/journal.pone.0185686

**Published:** 2017-10-31

**Authors:** Laura E. Lamb, Joseph J. Janicki, Sarah N. Bartolone, Kenneth M. Peters, Michael B. Chancellor

**Affiliations:** 1 Department of Urology, Beaumont Health System, Royal Oak, MI, United States of America; 2 Oakland University William Beaumont School of Medicine, Rochester Hills, MI, United States of America; 3 Underactive Bladder Foundation, Pittsburgh, PA, United States of America; Cedars-Sinai Medical Center, UNITED STATES

## Abstract

**Background:**

Interstitial cystitis/bladder pain syndrome (IC) is a multifactorial syndrome of severe pelvic and genitalia pain and compromised urinary function; a subset of IC patients present with Hunner’s lesions or ulcers on their bladder walls (UIC). UIC is diagnosed by cystoscopy, which may be quite painful. The objective of this study was to determine if a calculated Bladder Permeability Defect Risk Score (BP-RS) based on non-invasive urinary cytokines could discriminate UIC patients from controls and IC patients without Hunner’s ulcers.

**Methods:**

A national crowdsourcing effort targeted IC patients and age-matched controls to provide urine samples. Urinary cytokine levels for GRO, IL-6, and IL-8 were determined using a Luminex assay.

**Results:**

We collected 448 urine samples from 46 states consisting of 153 IC patients (147 female, 6 male), of which 54 UIC patients (50 females, 4 male), 159 female controls, and 136 male controls. A defined BP-RS was calculated to classify UIC, or a bladder permeability defect etiology, with 89% validity.

**Conclusions:**

The BP-RS Score quantifies UIC risk, indicative of a bladder permeability defect etiology in a subset of IC patients. The Bladder Permeability Defect Risk Score is the first validated urine biomarker assay for interstitial cystitis/bladder pain syndrome.

## Introduction

Interstitial cystitis/bladder pain syndrome (IC) is a chronic, severely debilitating disease of the bladder characterized by urinary frequency and urgency, and severe suprapubic, external genitalia, and/or pelvic pain [1]. IC severely compromises sexual function, employment, and quality of life. The estimated prevalence of IC in the U.S. is between 3–8 million women and 1–4 million men [2–4]. Approximately 10% of IC patients present with Hunner’s lesions or ulcers (UIC), distinct areas of inflammation on the bladder wall. UIC is usually associated with more severe symptoms and changes in the urothelium that suggest increased bladder permeability [5].

UIC can often be diagnosed by cystoscopy with hydrodistension demonstrating the distinct inflammatory lesions typically on the dome or lateral sidewalls of the bladder [6]. Cystoscopy can be painful in the patient with UIC. Most often, Hunner’s ulcers are identified during a diagnostic hydrodistention under general or regional anesthesia. Some patients may experience painful urination, hematuria, urinary tract infection, and rare risk of bladder wall perforation. Identifying and treating Hunner’s lesions allows the clinician to use bladder directed therapies such as electrocautery, resection, or injection of these lesions with steroids. In an effort to limit invasive testing and to meaningfully classify IC patients, we sought to determine if a urine based test could be developed to distinguish UIC from IC without Hunner’s lesions (NUIC). We focused on urinary cytokines given our past expertise and the standardized methodology [7].

Several groups, including ours, have previously published on several urine based proteins that are altered in IC and UIC. Many of these studies focused on IC and did not specifically include UIC. Several investigations have focused on increased activity of anti-proliferative factor (measured by inhibition of thymidine incorporation), increased epidermal growth factor, and decreased heparin-binding epidermal growth factor-like growth factor (HB-EGF) in IC [8–11]. Pro-inflammatory interleukin (IL)-6 has been reported to be increased in IC patients and has been positively associated with pain scores [8, 12–14]. IL-8 has also been reported in some studies to be increased in IC [8, 14]. Ogawa et al demonstrated differential expression of several mRNAs in the bladder urothelium of UIC compared to controls, including CXCR3 binding chemokine and TNFSF14 [15]. Recently, metabolite etiocholan-3α-ol-17-one sulfate (Etio-S) has been described as a potential IC biomarker for females [16]. Others have been reviewed by Kuo et al [17]. However, none of these possible biomarkers have been used successfully in the clinic thus far due to 1) overlap between controls, NUIC, and UIC and 2) difficulty of implementing the assay methodology [18]. Lastly, the urine samples for these studies were collected at academic centers where they could be immediately spun down and frozen for shipment and storage prior to analysis. This cold chain processing may not always be feasible, thus a solution where urine samples could be collected, shipped, and stored at room temperature without prior centrifugation would be ideal. There is currently no commercially available test for IC or UIC.

There are several innovations with this study. First, we validated a urine preservative method that allowed storage and shipment at ambient temperature. Second, we developed a crowdsource model in which participants collected and shipped urine samples to our laboratory toward joint development of an IC biomarker. Third, we used a machine learning method and developed a validated urine biomarker score based on a multiplex urine protein assay that provides UIC risk information independent of traditional clinical data.

## Methods

### Sample populations

The development of the BP-RS score used midstream urine samples that had a preservative (Norgen Biotek) added immediately after collection. For the IP4IC study, Norgen custom manufactured the preservative in a dried format into the bottom of a 100 mL urine collection cup so that the preservative would be immediately added to the urine during collection. The amount of powdered preservative was valid for 10-100mL of urine. All the urine samples collected for this study were in this range. Participants were instructed to invert the urine collection cup 10-20X until all the powdered preservative was dissolved. The urine was then shipped and stored at ambient temperature. For the P3 study, urine samples were kept on ice after collection and Norgen’s urine preservative in the liquid format (single dose ampule; Cat. No. 18124) was added to 25-50mL of urine within 30 min of collection and inverted 7X to ensure complete mixing. Samples were then stored at room temperature. The datasets (IP4IC and P3) used to develop and validate the final score algorithm respectively included samples from reported a) IC patients with Hunner’s lesions (UIC), b) IC patient’s without Hunner’s lesions (NUIC), and c) asymptomatic controls (control) subjects that were age matched. All participants were over 18 years of age. The Reporting recommendations for tumor MARKer prognostic studies (REMARK) were followed [19].

### IP4IC dataset

This study consisted of 448 urine samples from both IC and control participants collected through a crowdsourcing effort in collaboration with the Interstitial Cystitis Association (ICA). To evaluate in a more universal target population than just one geographical area and to collect a large cohort, we conducted a study on crowdsourced control, NUIC, and UIC samples termed IP4IC. These samples were collected by individuals in their homes and not in a clinical setting. Controls were age matched.

Study eligibility included a United States mailing address, and the ability and willingness to provide a urine sample and return it by mail. Exclusion criteria were urinary tract infection or any surgical prostate therapies (biopsies, microwave, needle ablation, balloon dilation, laser procedure, cryosurgery) within the last three months, or pregnancy. IC participants reported a physician diagnosed case for over 6 months. IC participants were also asked if a doctor had told them they have bladder ulcers, Hunner’s ulcers, Hunner’s lesions, or Hunner’s patches. Participants were asked to complete a short demographic survey, the Interstitial Cystitis Symptoms Index (ICSI) and Problem Index (ICPI), and a voiding diary. This was optional for controls. Participants were excluded from the study if they did not supply a urine sample.

### P3 dataset

51 midstream urine samples from IC and control participants were collected at Beaumont Hospital in Royal Oak, Michigan. All participants provided written consent. IC participants had a clinical history of IC. Asymptomatic controls had no history of IC, recurrent urinary tract infection, bladder or prostate cancer, or kidney disease. Exclusion criteria were unable to complete questionnaires or unable to provide urine specimen. Participants were asked to complete a short demographic survey, the ICSI, and the ICPI.

### Measurement of cytokines in urine samples

Expression of a panel of cytokines, chemokines, and growth factors was determined in urine samples using the commercially available MILLIPLEX MAP Human Cytokine/Chemokine Multiplex Immunoassay (Millipore) following manufacturer’s protocol and detailed in our previous publication [7]. No processing of urine samples was required; 25µL urine sample per well were taken from the middle part of the urine collect cup where it was free from any floating particles or sediment. All samples were run in duplicate or triplicate. Proteins tested included growth-related oncogene (GRO-α/CXCL1), IL-1 receptor antagonist (IL-1RA), Interleukin 6 (IL-6), Interleukin 8 (IL-8), interferon-inducible protein (IP-10/CXCL10), Monocyte chemoattractant protein-1/ (C-C Motif Chemokine Ligand 2 (MCP-1/CCL2), Regulated on Activation, Normal T Cell Expressed and Secreted (RANTES/CCL5), Vascular endothelial growth factor (VEGF), and platelet-derived growth factor (PDGF-BB) for the training data set. For the validation set and a portion of the training set, only GRO, IL-6, IL-8, and MCP-1 were measured. The assay range for all analytes was 3.2 to 10,000 pg/mL. The reported assay sensitivities for GRO, IL-6, and IL-8 was 9.9, 0.9, and 0.4 pg/mL respectively. 25 µL of undiluted urine was mixed with 25 µL of antibody conjugated microspheres (approximately 2,500 microspheres) per well and incubated at 4°C overnight (16–18 hours) in the dark on a plate shaker (500 rpm). An automated plate washer (Bio-plex Pro II, BioRad) was used for all wash steps. Fluorescence intensity of microspheres specific to each proteins of interest was measured within each sample simultaneously by an automated Bio-Plex® Luminex® 200 IS System immunoassay analyzer. The median fluorescent intensity was calculated and comparison to a standard curve using a 5-parameter logistic regression to calculate the concentrations of the samples. Controls and IC samples were analyzed on the same plate. Protein concentrations used were the average of technical duplicates. Each plate was run with a standard curve and two quality controls to qualify assay performance.

### IC prediction

Random Forest classification, a supervised machine learning method, was used to predict whether patients had Hunner’s lesions or ulcers (UIC). The method selects random subsets of data in a training set (IP4IC dataset), weighted appropriately according to the frequency of the different patient groups in this case, and builds decision trees. The decision trees are built by splitting the data in a way to minimize the gini impurity, a measure of classification accuracy. The classifier is trained with the levels of the three biomarkers, GRO, IL-6, and IL-8, and is labeled with the group to which a patient belongs (1 for UIC, and 0 for controls/NUIC). The biomarker training data are given as concentrations and are not normalized, since this generally degrades the performance of a random forest algorithm.

Performance and accuracy of machine learning methods is typically assessed with cross-validation. The Random Forest method, however, has an internal validation measure, called the out-of-bag (OOB) score. This score is continually calculated when building the trained classifier and updated by testing each decision tree on the data that was not included in the random subset used to build the tree. A higher OOB score corresponds to increased prediction accuracy.

Random Forests are parameterized, and a script was written to test combinations of reasonable parameters. A variety of parameters and ranges of values that were tested (Table 1). The Cartesian product (i.e. all combinations) of each value in all ranges of input variables were tested to train the classifier. Optimal results were determined (Table 2). The Python 3.6 language and Scikit-Learn v0.18 machine learning package were used to build the classifier.

**Table 1:** Parameter ranges for optimization of classifier.

Parameter	Description	Range
n_estimators	Number of decision trees in forest.	[10, 100], step = 1
criterion	Criteria by which to make a decision to split a tree node.	[‘gini’, ‘entropy’]
max_features	Number of features to consider when splitting data.	[1, 2, 3, $\sqrt{n\_features}$]
max_depth	Maximum depth of tree.	[None] (no limit imposed on tree depth)
min_samples_split	Minimum number of samples required to split an internal node.	[2]
min_samples_leaf	Minimum number of samples required to be at a leaf node.	[1,30], step = 1
min_weight_fraction_leaf	Minimum weighted fraction of the sum total of weights required to be at a leaf node.	[0]
max_leaf_nodes	Limits total number of leaf nodes.	[None] (no limit placed on total number of nodes)
bootstrap	Whether bootstrap samples are used when building trees.	[True]
oob_score	Whether OOB score is calculated when building trees.	[True]
n_jobs	Number of parallel jobs to run.	[–1] (uses all available processors/cores)
random_state	Seed value for random number generator. By default no random seed is set.	[42] (arbitrarily chosen so trees can be rebuilt if needed)
class_weight	Weights associated with each class (ulcerative IC = 1 or no IC/nonulcerative IC = 0) of data.	[‘balanced_subsample’] & splits of 0 = X%, 1 = Y% for (X in 10 -> 90, step = 10 and Y in 90 -> 10, step = 10)

**Table 2:** Optimal parameters that resulted in highest OOB score for training set.

Parameter	Value
n_estimators	22
criterion	‘gini’
max_features	$\sqrt{3}$
max_depth	None
min_samples_split	2
min_samples_leaf	3
min_weight_fraction_leaf	0
max_leaf_nodes	None
bootstrap	True
oob_score	True
n_jobs	-1
random_state	42
class_weight	‘balanced_subsample’

There were 16,000 total combinations tested in this optimization, with each classifier training taking about 5–15 seconds on an Intel Core i3-540 (3.06 GHz, dual core) processor. Further information about these parameters can be found in Pedregosa et al [20].

### Validation of the BP-RS score

The BP-RS score algorithm was validated in an independently conducted study, termed the P3 study. P3, another dataset collected in a similar manner to IP4IC but in the clinic, was used for external validation of the optimal trained classifier. This was used to assess if the accuracy of the classifier was reasonably close to the OOB score that was calculated. To test the classifier, the P3 training set was read into a program blinded, and the protein values were fed into the trained classifier. The protein data is run through the decision trees, and the average of the classes of all the decision trees is used to classify the patient. For example, if there are 15 of 22 total trees that classify the patient as having UIC, there is a 15 / 22 = 0.68 probability that the patient has UIC based on the constructed decision trees. Whenever this average probability is > = 0.5, the patient is predicted to have (i.e. is assigned the class of) 1 (UIC), otherwise it is assigned 0 (control or NUIC).

Patients were excluded from both the training and validation sets if protein data was incomplete (e.g. analyte detection was out-of-range). During collection of the IP4IC and P3 datasets, some values for protein levels were marked as being outside detection limits; patients missing data for even one of the protein analytes were excluded. Not all controls completed the questionnaires. As such, analysis of control questionnaires was based on the available data.

### Statistics

Cytokines were initially analyzed independently for statistically significant differences between UIC compared to NUIC and controls using Prism 6 software (GraphPad). This was done using Kruskal-Wallis non-parametric ANOVA. Two-tailed unpaired Mann-Whitney test was used for comparison of 2 groups. Results are expressed as mean ± SEM, and differences were considered significant at *p* < 0.05. ROC curve analysis was performed to evaluate the performance of the BP-RS in discriminating between UIC, NUIC, and control groups. ROC analysis plots sensitivity (true-positive rates) versus 1-specifity (false-positive rates). These curves were calculated separately for each dataset with the sklearn.metrics.roc_curve function from scikit-learn. The AUC is a measure of the discrimination power of the BP-RS. The AUCs for both data sets were calculated with the sklearn.metrics.roc_auc_score function.

### Study approval

All studies had approval from Beaumont Health System’s Institutional Review Board (IRB). For the IP4IC study (IRB approval #2015–323), an information sheet outlining the rights of the participant as it relates to the research and what study participation entails was provided to participants; full consent was provided by completing an online or mailed survey and submission of the urine specimen. The online survey for IC participants included two questions to confirm voluntary participation and desire to enroll in study before proceeding with the rest of the survey. Control participants were recruited by the IC participants and urine samples and surveys were filled out anonymously; no names or addresses were collected from controls at any point. For the P3 study (IRB approval #2014–281), written consent was obtained from all participants prior to inclusion in the study.

## Results

### Characteristics of study participants

**Fig 1:**
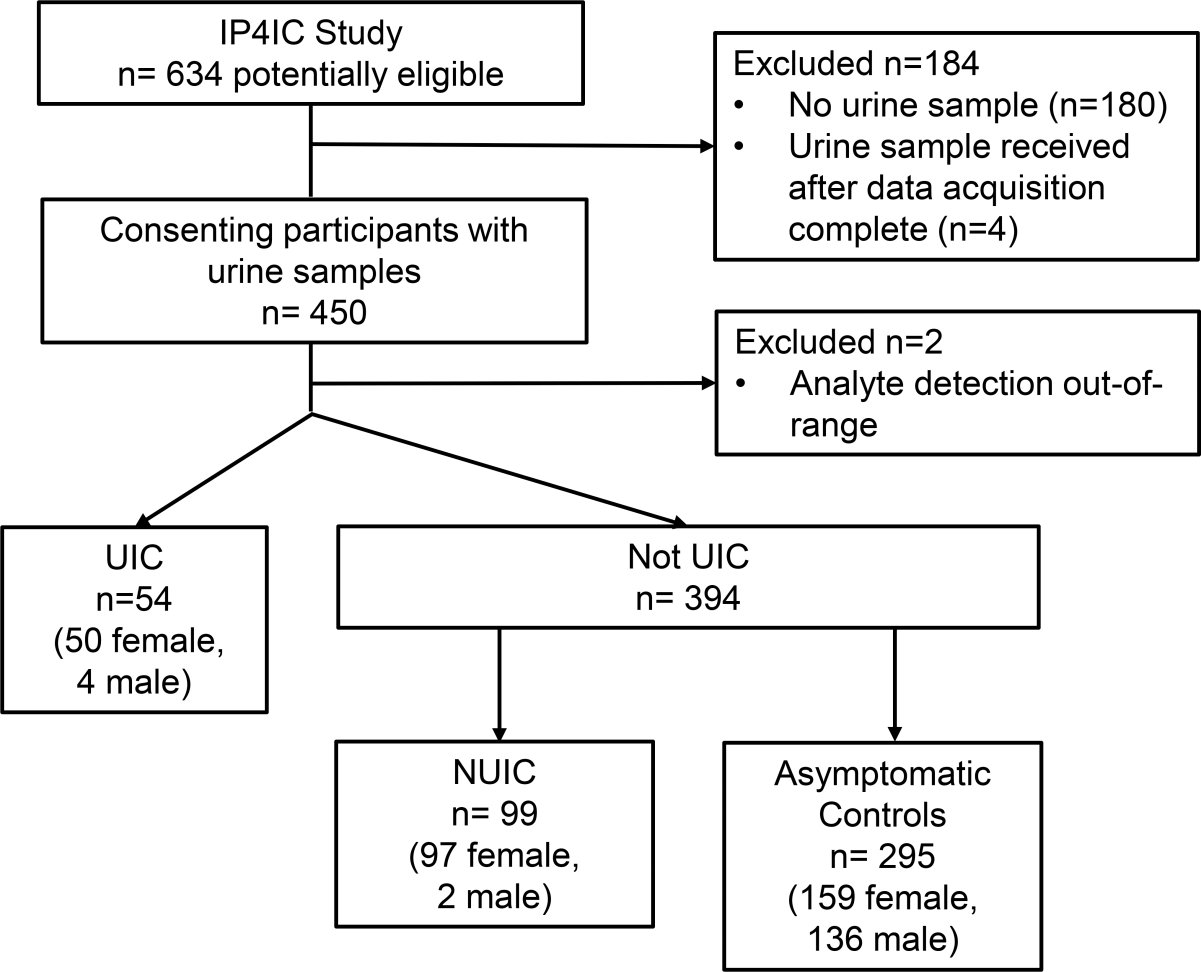
Consolidated standards of reporting trials flow diagram for study numbers. UIC = IC with Hunner’s lesions; NUIC = IC without Hunner’s lesions.

There were 454 total patients in the final IP4IC training dataset. The samples were sent by express mail at ambient temperature from 46 states in the US. Four samples were received after data acquisition had been completed and not included in the training set; only patients with urine samples were included in the study. Two samples during data acquisition had analyte measurements that were larger than the upper detection limit and were excluded (Fig 1). Of the remaining 448 patients, 54 patients had IC with Hunner’s lesions and 394 had either no IC or IC without Hunner’s lesions. IC patients are primarily women and this was reflected in our study population (147 women out of 153 IC patients, or 96.1%). As expected, IC patients reported a higher mean interstitial cystitis symptom index (ICSI) of 14.8 ± 0.6 for UIC participants and 11.5 ± 0.5 for NUIC participants, compared to asymptomatic controls at 3.0 ± 0.2 (Table 3). IC participants also reported higher interstitial cystitis problem index of 12.2 ± 0.5 for UIC and 9.8 ± 0.4 for NUIC compared to controls at 1.5 ± 0.1.

**Table 3:** Participant demographics.

Metric	IP4IC			P3		
	UIC	NUIC	Controls	UIC	NUIC	Controls
	(n = 54)	(n = 99)	(n = 295)	(n = 8)	(n = 17)	(n = 26)
Age (years)	54.4 ±1.7	50.8±0.3	50.5±0.8	61.1±5.1	62.9±3.3	56.6±3.6
Duration of symptoms (mos)	185±13.7	120.1±9.2	n/a	167.4±33.2	236.2±42.2	n/a
IC symptom index (0–20)	14.8±0.6	11.5±0.5	3.0±0.2	14.9±1.3	12.7±0.6	2.0±0.4
IC problem index (0–16)	12.2±0.5	9.8±0.4	1.5±0.1	12.9±1.2	11.5±0.5	1.5±0.9
# AM voids	8.4±0.9	5.5±3.9	2.8±1.3	No voiding diary collected		
# PM voids	10.2±1.3	7.3±4.8	3.8±1.9			
Total /24 h	18.6±2.1	12.8±8.3	6.7±2.8			
Leakage #	1.8±0.9	0.9±2.8	0.0±0.6			
Pain #	9.4±2.4	3.9±7.6	0.0±0.3			
Avg. AM Pain	5.1±0.4	3.7±2.9	0.1±0.5			
Avg. PM Pain	5.5±0.4	4.0±2.9	0.1±0.7			
Avg. AM Urgency	6.7±0.4	4.3±3.0	1.4±20.5			
Avg. PM Urgency	6.7±0.4	4.3±3.0	1.1±1.8			

### Urinary cytokines GRO, IL-6, and IL-8 are elevated in UIC patients compared to controls and NUIC patients

**Fig 2:**
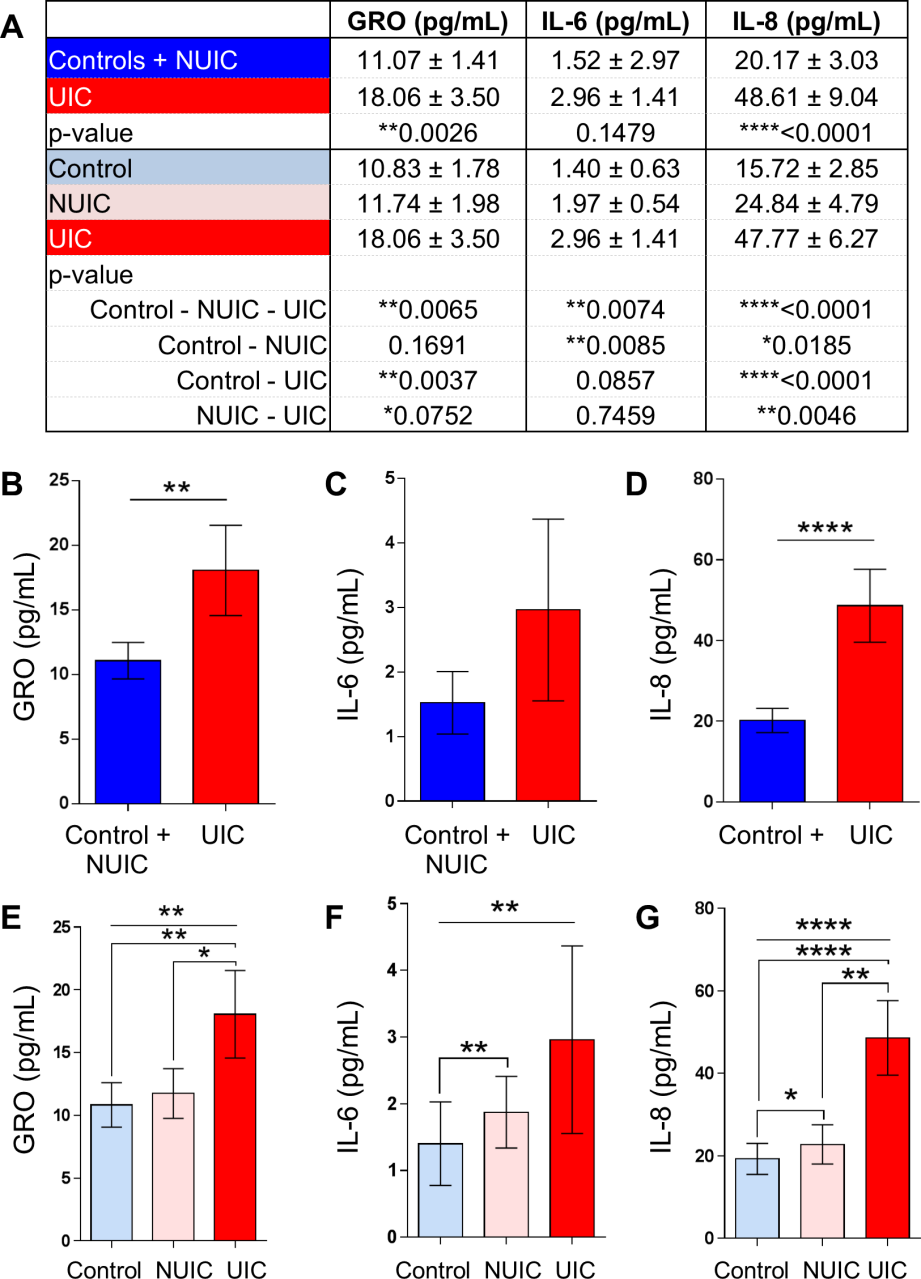
Urinary cytokine levels by group. **A)** Mean and SEM values for urine cytokines GRO, IL-6, and IL-8. **B-G)** Urinary levels of cytokines GRO, IL-6, and IL-8 in groups Control and NUIC compared to UIC **(B-D)** or control compared to NUIC compared to UIC **(E-F)**. Error bars are SEM. Statistical differences were determined using Mann-Whitney test **(B-G)** or Kruskal-Wallis ANOVA **(E-G)**. * indicates p<0.05, ** indicates p<0.01, *** indicates p<0.001, **** indicates p<0.0001.

The first step in developing the BP-RS was to determine the urinary expression levels of a panel of cytokines (Fig 2). Ideally, we wanted to develop a test that could discriminate UIC from both healthy asymptomatic controls and NUIC patients. Urinary GRO/CXCL-1 and IL-8 were significantly increased 1.63 fold and 2.41 fold respectively in UIC participants compared to the control and NUIC group (Fig 2A–2D). IL-6 was also elevated, but was not significant. We then compared controls to NUIC to UIC for urinary cytokine expression, in which there was a significant difference across groups for GRO, IL-6, and IL-8 (Fig 2A and 2E–2G).

### Development of BP-RS score

**Fig 3:**
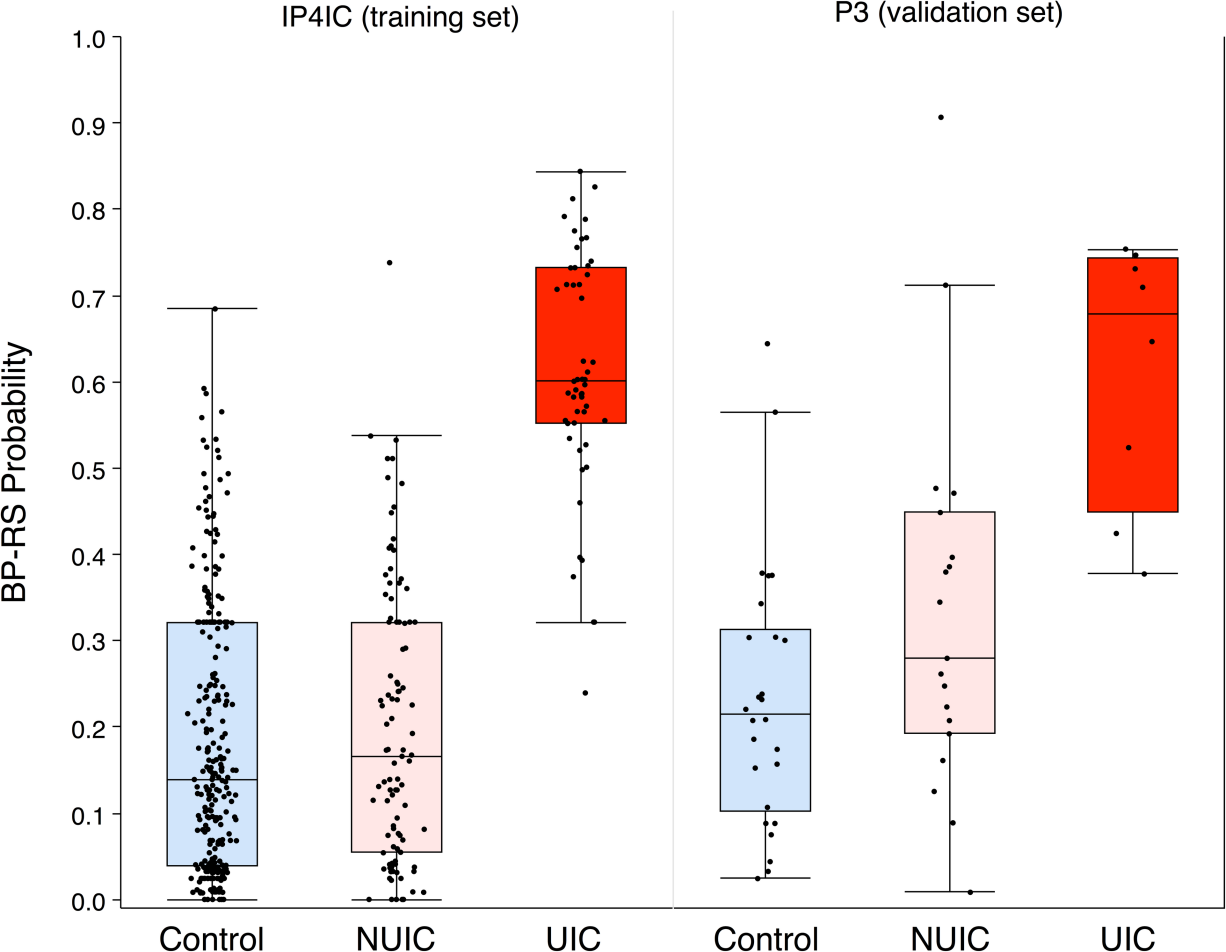
Average BP-RS probabilities in IP4IC training set and of P3 validation set. The BP-RS is a score that gives the probability that a patient has UIC. Each of the validation data points were fed into the trained Random Forest Classifier (RFC), and the BP-RS (probability of UIC) was calculated and plotted for each patient. The bars on the boxes describe the minimum and maximum points, and the horizontal lines of the box show the 25%, 50% (median), and 75% quartiles. Points outside of the bars are outliers. The individual probabilities are also plotted (black points). Because we used a binary classifier, all of the points above 0.5 (dotted line) are predicted to have UIC, and all points below are classified as control or NUIC. In the validation set, two patients from each of the control and NUIC groups were incorrectly classified as having UIC, and two points from the UIC group were incorrectly classified as not having UIC.

Although UIC urine samples had higher expression of GRO, IL-6, and IL-8, there was overlap in expression across controls, NUIC, and UIC (Fig 2). Measurement on any of these cytokines alone was insufficient to classify a patient into any of these groups. We therefore sought to develop a supervised machine learning method using Random Forest Classification (RFC) for predicting if a person had UIC based on information from urinary expression of more than one cytokine. RFC has been demonstrated to be a superior classifier to distinguish disease from non-disease biological samples in previous studies, which extensively compared RFC to other approaches including k-nearest-neighbor approach (KNN), linear discrimination analysis (LDA), small vector machine (SVM), and others methods [21–23]. RFC utilizes random sampling and ensemble strategies that have several advantages including good predictive or classifying accuracy, ability to analyze a mixture of variable types (continuous, binary, and categorical), does not require fine-tuning of parameters or pre-selection of variables to eliminate noise, RFC measures variable importance, it does not overfit data, it can handle complex data structures, and lastly RFC produces smaller biomarker sets compared to other machine learning methods [21–23]. RFC Classifiers were built and tested using either one (e.g. GRO, IL-6, or IL-8 alone) or combinations of two of these biomarkers (e.g. GRO + IL-6, GRO + IL-8, IL-6 + IL-8) using the same parameters, and performance was worse than random guessing (OOB < 50%). The same optimization procedure in Table 1 was also applied to each of these sets individually, which resulted in OOB scores between 40–60%. However, only the combination of all three biomarkers (GRO + IL-6 + IL-8) led to reasonable prediction capabilities (OOB = 81.5%). The final classifier with optimized parameters was termed the Bladder Permeability Defect Risk Score, or BP-RS (Fig 3).

Using the random forest classifier method, the relative importance of each cytokine used to make a classification is evident from the composition of the decision trees. IL-8 contributed most significantly toward the predictions (48%), followed by GRO (33%) and IL-6 (19%), which made smaller, but still significant contributions.

### Validation of BP-RS score

**Fig 4:**
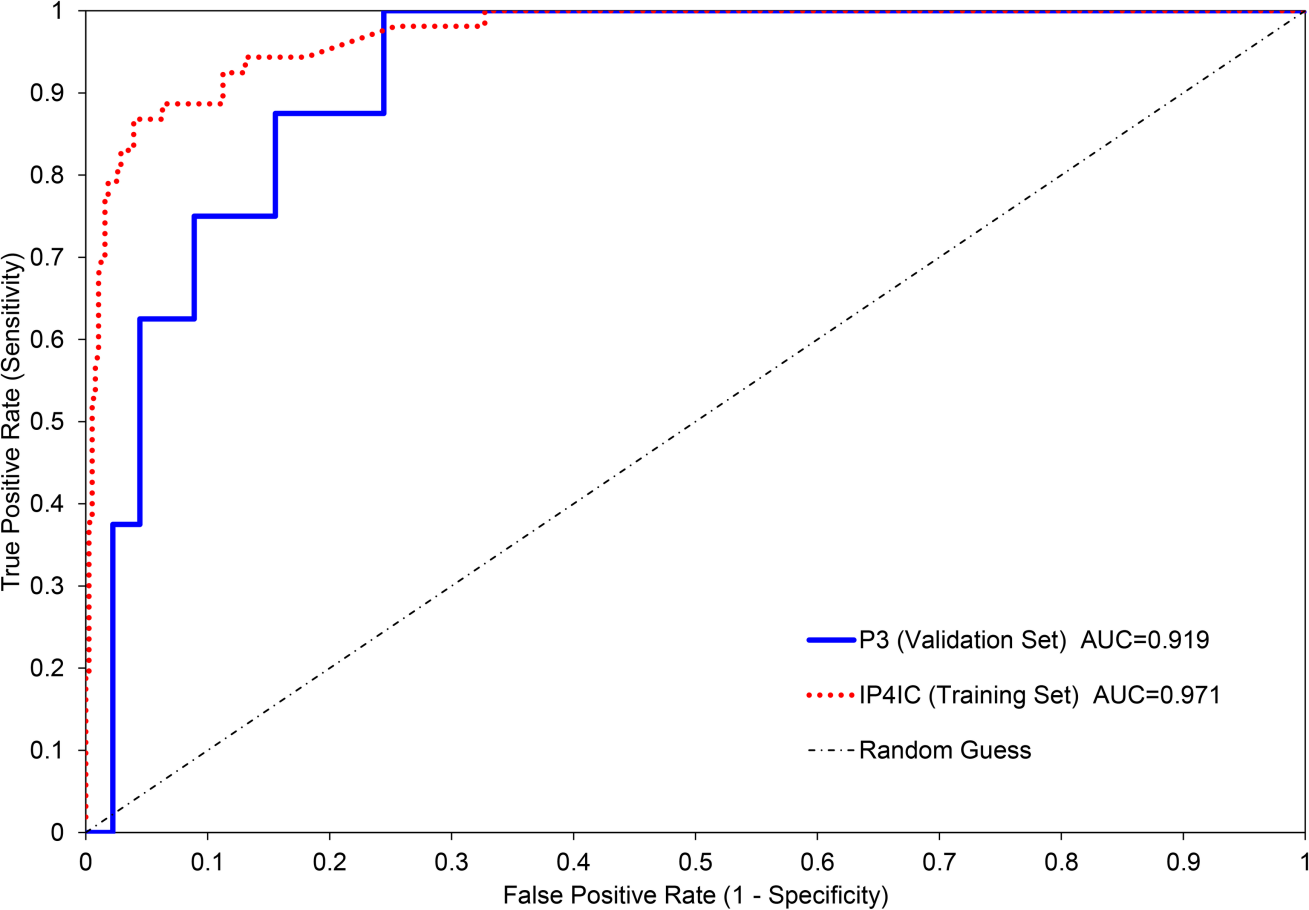
Receiver operating characteristic (ROC) curves. ROC curves for the IP4IC (training set) and P3 (validation set) using the BP-RS. The AUC for the IP4IC dataset = 0.971 and P3 dataset = 0.919.

Internal validation, by an out-of-bag (OOB) score, was used to measure the prediction error of the BP-RS. A higher OOB score corresponds to increased prediction accuracy. The highest OOB score for the trained classifier with optimal parameters was found to be 81.5%. To further assess the accuracy of the BP-RS in predicting UIC, we performed receiver operating characteristic (ROC) analysis (Fig 4). The area under the curve (AUC) for the IP4IC dataset = 0.971 and P3 dataset = 0.919. Since the IP4IC data was used to train the classifier model, the high AUC value and curve shape here is expected. The ROC generated from the P3 validation set has a lower, but still high AUC, indicating that there may be reasonable diagnostic utility of the trained classifier (Fig 4). Furthermore, the AUC value for the P3 validation set had poor performance when only one or two biomarkers were used (S1 Table). A combination of all three biomarkers was required for a high AUC for the P3 validation set. The external validation set, P3 (n = 53), consisted of 8 UIC patients and 45 patients that were either NUIC or controls. 75% (6/8) of the UIC patients were correctly predicted to have UIC (S2 Table). 91.1% (41/45) of patients with NUIC or controls were correctly predicted, which resulted in 4 patients with false-positive predictions for UIC. Upon referencing these patients with the dataset, it was found that two of these patients had NUIC and two were controls. The IP4IC training set and P3 validation set had a false positive rate of 2.15% and 4.65% respectively, a negative predictive value of 96.0% and 93.2% respectively, and a positive predictive value of 84.9% and 75.0% respectively (S2 Table). A reasonable false positive rate, negative predictive value, and positive predictive value were only obtained for the P3 validation set when all three biomarkers were used (S2 Table). Overall, 88.7% of the entire validation set was correctly predicted. This result can be expected to converge to the OOB score if more validation test samples were present, and it may be possible to increase the OOB score with an expanded training set or an expanded parameter search.

## Discussion

We hereby demonstrate that applying a machine learning classification model of a combination of three urinary cytokines we can develop a novel score, the BP-RS, which can distinguish IC with Hunner’s lesions from IC without Hunner’s lesions or controls. It has been proposed previously that IC with or without Hunner’s lesions may have distinct pathophysiologies [17], and that IC with Hunner’s lesions may have a more bladder-centric involvement whereas IC without Hunner’s lesions may be more systemic. Patients with NUIC can have other comorbid pain syndromes, such as pelvic floor dysfunction, and respond better to systemic therapy. In contrast, UIC respond to more localized therapy focused on the bladder with urothelial permeability defect. As such, UIC and NUIC may have different disease etiologies and therefore differential biomarkers may be feasible.

We developed a process by which patients can collect urine in their own homes and then ship the samples to a collection facility/laboratory to be stored at ambient temperature until analyzed at a later date. Urinary cytokines are measured by Luminex xMAP technology, and a Random Forest Classification model is applied to the raw data, which provides an IC with Hunner’s lesions prediction score for each patient. We believe this is the first evidence that preserved urine stored and shipped at ambient temperature can be successfully used to predict UIC from both controls and NUIC. This is through the unique use of a classification model that utilizes a combination of three cytokines, IL-6, IL-8, and CXCL-1/GRO.

Most biomarker testing using urine samples has focused on using frozen urine samples. Typically this requires urine samples to be placed on ice or refrigerated immediately after collection, spun down in a refrigerated centrifuge to remove cellular debris, then stored at -80°C until use. The thawed sample may be spun down again before data acquisition. This can be challenging in that the samples must remain cold at all times, and maintaining this cold chain may not be possible or feasible in all settings. Also, the stability of proteins can be affected by freeze-thaw cycles or processing delay [24, 25]. The use of a urine preservative circumvents the need for a cold chain from sample collection to analysis in addition to inhibiting bacteria and fungi growth, allowing samples to be shipped at ambient temperatures.

We used Random Forest Classification to develop the BP-RS score. RFC provides prediction accuracy and model interpretability without a need to fine-tune parameters, as may be required by other machine learning methods. RFC is an increasingly popular approach to deal with complex biological data, including classification of samples based on genetic or proteomic expression [21]. The BP-RS required all three proteins (GRO, IL-6, and IL-8) to properly classify a patient’s risk for UIC; using one protein or two proteins in combination was insufficient. The validation set (P3) has the best performance when all three biomarkers are used for the area under the curve, false positive rate, negative predictive value, and positive predictive value. The model presented here was trained on the IP4IC dataset, but an advantage of using RFC is that it can be regenerated and improved when data is obtained from new patients outside the dataset. Additional training data generally results in better prediction accuracy, and will likely change the optimal parameters of the RFC.

Our model demonstrates that differences in UIC compared to NUIC and controls are reflective of urinary expression of GRO, IL-6, and IL-8. This may represent a distinct bladder permeability defect etiology in UIC bladders. IL-8 contributed most significantly toward the predictions (48%), followed by GRO (33%) and IL-6 (19%). Interestingly, IL-8 and GRO are both similar in protein structure and can be potent chemoattractants for neutrophils and basophils and promoters of angiogenesis, with IL-8 being the stronger chemoattractant of the two [26]. Both infiltration of mononuclear cells and angiogenesis are strongly associated with Hunner’s lesions in IC [27, 28]. Although several groups have investigated the expression of IL-8 in IC [8, 14], GRO has been less studied. IL-6 also is a pro-inflammatory cytokine and has been previously reported to be elevated in IC [8, 12–14]. It has also been reported to be increased in other urological disease, such as bacterial cystitis and bladder cancer [29–31]. It had the lowest expression in UIC of the three cytokines, and contributed the least to the BP-RS. IL-6 expression may be reflective of general inflammation.

To collect samples that would represent the spectrum of IC disease across the country, we developed a crowdsource biomarker development model in which participants from 46 states in the US collected and shipped urine samples to our laboratory at ambient temperature via express mail. As the urine samples were collected at home and not in a clinical setting, this introduced some risk of non-standardized sample collection and handling. We took several steps to minimize possible errors. This included both written instructions contained in the collection kit, as well as online written and video tutorials on how to collect and ship samples (https://www.**youtube**.com/watch?v=GL9T7ISyImk). Participants were also provided an email address and phone number they could use if they had any questions. Lastly, the urine preservative was manufactured as a powder located on the inside of the collection cup, so there were no variations in the time between urine collection and addition of preservative. Overall, this demonstrates patient compliance and downstream testing feasibility in collecting urine samples at home and shipping back for laboratory testing.

In this study, the majority of the IC patients were female (146/153; 95.4%) which is similar to what is found clinically. However 45.9% of our control participants were male (130/283). This did not influence the results as female and male control participants had similar BP-RS value and distribution.

One limitation of the study is that UIC and NUIC classifications were self-reported by the patient for the IP4IC data set and we were not able to confirm these designations in a clinical setting. Therefore it is possible that some patients may be misclassified according to current National Institute for Diabetes and Digestive and Kidney Diseases criteria [32]. However, self-reported patient disease designations have been shown to be sensitive and are commonly used in disease surveillance [33]. However, the P3 data set was able to be clinically confirmed. Our study was limited to one urine collection and to participants with a United States address. Future studies will follow BP-RS scores and symptoms in individuals over time, investigate if treatments impact the BP-RS in UIC patients, and determine if the BP-RS correlates with ICSI or ICPI scores. We may also investigate other urological diseases, such as bladder and prostate cancer, to determine the specificity of BP-RS.

In conclusion, this study demonstrates that BP-RS can classify UIC probability based on urinary cytokines using preserved urine samples collected, shipped, and stored at ambient temperature. We achieve diversity in sample source collection from across the US and engaged the national IC community through a novel crowdsourcing process toward joint disease biomarker development. The BP-RS Score provides a new clinical and research tool to phenotype IC patients without need for hydrodistention and cystoscopy, or a cold-chain from urine sample collection to data acquisition. It also demonstrates the utility of multiplex assays and random forest classification for development of novel and unique scores to classify patients with complex and overlapping urinary proteomic expression. The Bladder Permeability Defect Risk Score is the first validated urine biomarker assay for interstitial cystitis/bladder pain syndrome.

## Supporting information

S1 Table Area under the curve for all biomarker combinations tested. (DOCX)

S2 Table False positive rate, negative predictive value, and positive predictive value for all biomarker combinations tested. (DOCX)
