# Supplementary material for: Development of an interstitial cystitis risk score for bladder permeability
Source: PLoS One. 2017 Oct 31;12(10):e0185686. doi: 10.1371/journal.pone.0185686 (PMC5663335; doi:10.1371/journal.pone.0185686)
Supplement: S1 Table — (DOCX) [file pone.0185686.s001.docx]

**S1 Table. Area Under the Curve for All Biomarker Combinations Tested**

| **Biomarker(s)** | **AUC** | |
| --- | --- | --- |
|  | **IP4IC**  **(training set)** | **P3  (validation set)** |
| GRO | 0.871 | 0.315 |
| IL-6 | 0.729 | 0.513 |
| IL-8 | 0.932 | 0.448 |
| GRO & IL-6 | 0.915 | 0.350 |
| GRO & IL-8 | 0.979 | 0.478 |
| IL-6 & IL-8 | 0.938 | 0.433 |
| IL-6 & IL-8 & GRO | 0.971 | 0.919 |
