# Supplementary material for: Development of an interstitial cystitis risk score for bladder permeability
Source: PLoS One. 2017 Oct 31;12(10):e0185686. doi: 10.1371/journal.pone.0185686 (PMC5663335; doi:10.1371/journal.pone.0185686)
Supplement: S2 Table — (DOCX) [file pone.0185686.s002.docx]

**S2 Table. False Positive Rate, Negative Predictive Value, and Positive Predictive Value for All Biomarker Combinations Tested**

| **Biomarker(s)** | **False Positive Rate** | | **Negative Predictive Value** | | **Positive Predictive Value** | |
| --- | --- | --- | --- | --- | --- | --- |
|  | **IP4IC  (training set)** | **P3  (validation set)** | **IP4IC  (training set)** | **P3  (validation set)** | **IP4IC  (training set)** | **P3  (validation set)** |
| GRO | 0.108 | 0.265 | 0.944 | 0.857 | 0.446 | 0.0714 |
| IL-6 | 0.0737 | 0.265 | 0.912 | 0.857 | 0.404 | 0.0714 |
| IL-8 | 0.0974 | 0.347 | 0.961 | 0.865 | 0.513 | 0.105 |
| GRO & IL-6 | 0.0579 | 0.224 | 0.950 | 0.884 | 0.607 | 0.154 |
| GRO & IL-8 | 0.0347 | 0.286 | 0.976 | 0.854 | 0.772 | 0.0667 |
| IL-6 & IL-8 | 0.0684 | 0.347 | 0.967 | 0.842 | 0.612 | 0.0556 |
| IL-6 & IL-8 & GRO | 0.0215 | 0.0465 | 0.960 | 0.932 | 0.849 | 0.750 |
